# Autoantibodies May Predict Immune-Related Toxicity: Results from a Phase I Study of Intralesional *Bacillus Calmette–Guérin* followed by Ipilimumab in Patients with Advanced Metastatic Melanoma

**DOI:** 10.3389/fimmu.2018.00411

**Published:** 2018-03-02

**Authors:** Jessica Da Gama Duarte, Sagun Parakh, Miles C. Andrews, Katherine Woods, Anupama Pasam, Candani Tutuka, Simone Ostrouska, Jonathan M. Blackburn, Andreas Behren, Jonathan Cebon

**Affiliations:** ^1^Olivia Newton-John Cancer Research Institute, Heidelberg, VIC, Australia; ^2^School of Cancer Medicine, La Trobe University, Heidelberg, VIC, Australia; ^3^Ludwig Institute for Cancer Research, Melbourne-Austin Branch, Melbourne, VIC, Australia; ^4^MD Anderson Cancer Center, University of Texas, Houston, TX, United States; ^5^Department of Integrative Biomedical Sciences and Institute of Infectious Disease and Molecular Medicine, University of Cape Town, Cape Town, South Africa; ^6^Sengenics Corporation, Singapore, Singapore

**Keywords:** melanoma, *bacillus Calmette–Guerin*, ipilimumab, immune-related adverse events, protein microarrays

## Abstract

Immune checkpoint inhibitors (ICIs) have revolutionized the treatment of advanced melanoma. The first ICI to demonstrate clinical benefit, ipilimumab, targets cytotoxic T-lymphocyte-associated antigen-4 (CTLA-4); however, the long-term overall survival is just 22%. More than 40 years ago intralesional (IL) *bacillus Calmette–Guérin* (BCG), a living attenuated strain of *Mycobacterium bovis*, was found to induce tumor regression by stimulating cell-mediated immunity following a localized and self-limiting infection. We evaluated these two immune stimulants in combination with melanoma with the aim of developing a more effective immunotherapy and to assess toxicity. In this phase I study, patients with histologically confirmed stage III/IV metastatic melanoma received IL BCG injection followed by up to four cycles of intravenous ipilimumab (anti-CTLA-4) (ClinicalTrials.gov number NCT01838200). The trial was discontinued following treatment of the first five patients as the two patients receiving the escalation dose of BCG developed high-grade immune-related adverse events (irAEs) typical of ipilimumab monotherapy. These irAEs were characterized in both patients by profound increases in the repertoire of autoantibodies directed against both self- and cancer antigens. Interestingly, the induced autoantibodies were detected at time points that preceded the development of symptomatic toxicity. There was no overlap in the antigen specificity between patients and no evidence of clinical responses. Efforts to increase response rates through the use of novel immunotherapeutic combinations may be associated with higher rates of irAEs, thus the imperative to identify biomarkers of toxicity remains strong. While the small patient numbers in this trial do not allow for any conclusive evidence of predictive biomarkers, the observed changes warrant further examination of autoantibody repertoires in larger patient cohorts at risk of developing irAEs during their course of treatment. In summary, dose escalation of IL BCG followed by ipilimumab therapy was not well tolerated in advanced melanoma patients and showed no evidence of clinical benefit. Measuring autoantibody responses may provide early means for identifying patients at risk from developing severe irAEs during cancer immunotherapy.

## Introduction

The treatment of metastatic melanoma is rapidly evolving with the approval of a number of targeted therapies and immune checkpoint inhibitors (ICIs) in a short period of time. Despite significant improvement in outcomes, the median overall survival of patients with advanced disease remains poor (1). Ipilimumab, a fully humanized IgG1 monoclonal antibody, is the first ICI to show a survival benefit in advanced melanoma in treatment-naïve and pretreated patients (2). Ipilimumab competitively binds to cytotoxic T-lymphocyte-associated antigen-4 (CTLA-4) more efficiently than B7 molecules found on antigen-presenting cells, preserving CD28 signaling to potentiate antitumor T-cell responses. Side effects from ipilimumab, called immune-related adverse events (irAEs), are typically inflammatory in nature and may relate to the activation of the immune system against self-antigens (3). High-grade 3 or 4 irAEs occur in 10–15% of patients: primarily colitis, diarrhea, rash, hepatotoxicity, and endocrinopathies (4, 5).

*Bacillus Calmette–Guérin* (BCG) is a living attenuated strain of *Mycobacterium bovis* that stimulates cell-mediated immunity by producing a localized and self-limiting infection. It has been shown to have antitumor activity in several clinical studies (6–9). The exact mechanism of action is not well known, but it is probable that BCG invokes a local inflammatory response involving a variety of both innate and adaptive immune effector cells (10). Intravesical immunotherapy with BCG has been established as the most effective adjuvant treatment for preventing local recurrences and tumor progression following transurethral resection of nonmuscle invasive bladder cancer. A large number of clinical trials have established a major role for BCG immunotherapy in urological oncology (11–13).

Intralesional (IL) BCG can be effective in inducing the regression of cutaneous metastatic melanoma (7, 9, 10, 14, 15). Inflammation and ulceration occurred in most cases, and subsequent regression of the injected lesion was commonly observed. In fewer than 10% of patients receiving IL BCG, regression of noninjected lesions was seen, and occasional long-term disease-free survival has been reported (15, 16), likely due to persistent T-cell immunity. Side effects were dose-dependent and included largely constitutional flu-like symptoms such as fever and myalgia, generally lasting 8–9 weeks that could be stopped with isoniazid (17).

The safety and efficacy of BCG given in combination with melanoma vaccines were evaluated in several phase I, II and III clinical trials (9, 18–20). The multicenter phase III randomized studies of BCG plus a polyvalent melanoma vaccine (CancerVax) versus BCG plus a placebo as a postsurgical treatment for stage III or IV melanoma (MMAIT-III and MMAIT-IV trials) were stopped when interim analyses demonstrated that it was unlikely that the vaccine would provide significant evidence of a survival benefit. Nevertheless, excellent survival was seen for the entire study population with 42% of stage IV and 63% of stage III patients projected to be alive at 5 years (21). This high survival may have been due to selection bias or BCG, which may have acted as an active immunotherapeutic agent at the administered dose.

Despite the success of ICI, a significant proportion of patients either does not respond to treatment or becomes resistant after initial response. This failure of therapy may result from a variety of mechanisms, such as immune ignorance, a hostile tumor environment, alternative immune checkpoint-independent regulatory mechanisms, inadequate antigenicity, or antigen downregulation (22). Strategies that induce a favorable inflammatory tumor microenvironment prior to, or at the time of, ICI have the potential to increase the effectiveness of anticancer immune therapies.

In this study, we evaluated the safety, clinical efficacy, and immunogenicity of IL BCG followed by ipilimumab (supported by the Ludwig Institute for Cancer Research and by Bristol-Myers Squibb; ClinicalTrials.gov number NCT01838200/LUD2012-003). Given the possibility of ipilimumab to potentiate the inflammatory effects of BCG, particular attention was paid to the evaluation of local and systemic inflammatory toxicities.

Protein microarrays have been widely used to detect and quantify the presence of autoantibodies in a variety of autoimmune diseases (23). Since patients treated with immunotherapy develop irAEs that resemble autoimmune disease, we further investigated the autoantibody repertoire of all recruited patients to characterize their serological responses as part of our broader immune-monitoring approach.

## Materials and Methods

### Patients

Patients of good performance status (Eastern Cooperative Oncology Group score 0–1) with histologically confirmed unresectable stage III or stage IV melanoma were enrolled in the study. The major inclusion criteria included the presence of at least one cutaneous or subcutaneous metastatic lesion amenable to IL therapy. Key exclusion criteria included symptomatic or active cerebral metastases requiring corticosteroids, prior history of tuberculosis, hypersensitivity to BCG or contraindication to the use of isoniazid, autoimmune disease, immunodeficiency disease, or the use of immunosuppressive therapy.

### Study Design

**Figure 1:**
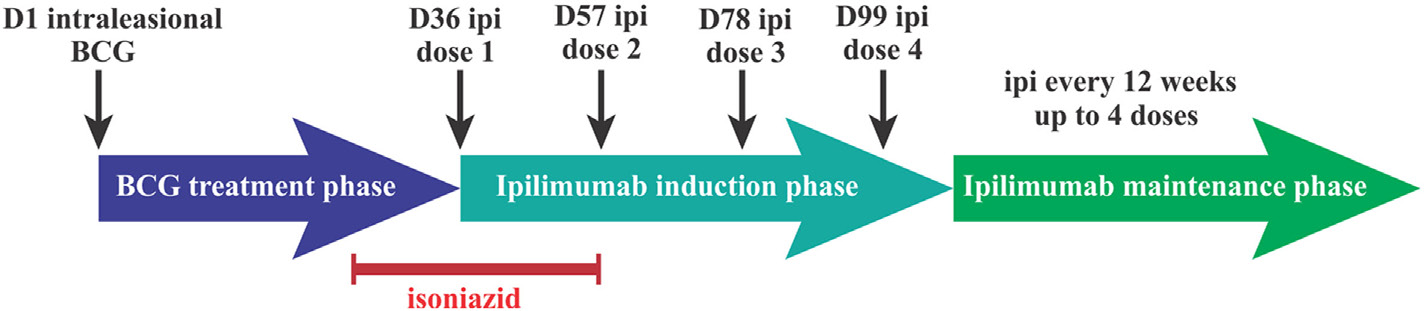
Intralesional *bacillus Calmette–Guérin* (BCG) followed by ipilimumab phase I trial treatment schedule. Dn, day n; Ipi, ipilimumab.

This was a single-site, open-label phase I, dose-escalation study. Because of the potential for ipilimumab to amplify inflammatory responses mediated by BCG, a variety of safety precautions were taken; eligible patients had a skin test for tuberculin reactivity with purified protein derivative and were enrolled in one of two cohorts, depending on the size of the induration. Patients with an induration of <10 mm in diameter were enrolled in cohort 1 which utilized a 3 + 3 dose-escalation design. Patients with a reaction of ≥10 mm were enrolled in cohort 2. Enrollment of the first three patients was staggered by 3 weeks; subsequent patients were enrolled without delay. Patients enrolled in cohort 1, group 1, received 200 µl BCG (day 1, D1) containing 0.16–0.64 × 10^6^ cfu, and patients in groups 2 and 3 received 200 µl BCG containing 0.80–3.20 × 10^6^ cfu and 4.00–16.00 × 10^6^ cfu, respectively. All patients enrolled in cohort 2 received 200 µl BCG containing 0.16–0.64 × 10^6^ cfu BCG. To ensure that no active BCG infection was present at the time of ipilimumab administration, oral isoniazid of 300 mg/day was commenced on D29 and continued for 4 weeks in all patients. Ipilimumab was administered intravenously on D36 at a dose of 3 mg/kg every 3 weeks for a total of four doses. Patients with responding or a stable disease by RECIST v.1.1 or immune-related response criteria (irRC) were scheduled to receive ipilimumab as maintenance therapy administered at a 12-week interval for a further four doses (Figure 1).

This study was conducted at Austin Health, Heidelberg, Australia, and was approved by the institution’s human research ethics committee. Written informed consent was obtained from all patients, and all methods were performed in accordance with the protocol-specified guidelines and regulations.

### Dose-Limiting Toxicity (DLT)

Dose-limiting toxicity (DLT) was defined as ≥grade 3 skin reaction at the injection site or ≥grade 3 toxicity associated with BCG administration. All toxicities were graded as per the Common Terminology Criteria for Adverse Events (CTCAE) version 4.0. The protocol included provisions for BCG dose adjustments in the event of grade 3 or 4 toxicities including local skin reactions. DLT of ipilimumab was defined as any toxicity that required dosing modifications in accordance with the recommendations in the product information, or ≥grade 3 hematologic or nonhematologic toxicity.

### Safety Assessments: Screening, Baseline, and Follow-up

Safety assessments were carried out at patient enrollment, D1, D29, D36, D43, D50, D57, D78, D99, D113, D141, and D204. Target skin lesions were monitored with clinical photography at D1, D36, D113, D141, and D204. Plasma and peripheral blood mononuclear cells were collected at D1, D36, D43, D57, D78, D113, D141, and D204.

### Response Assessment

Tumors were assessed clinically and by contrast-enhanced (CT) scans (brain, neck, chest, abdomen, and pelvis) at patient enrollment, D36, D113, D141, and D204. Tumor response and progression were determined in accordance with RECIST v1.1 criteria (24) and irRC (25).

### Autoantibody Profiling Using the Immunome™ Protein Arrays

A total of 15 plasma samples were assayed using the Immunome™ protein array (Sengenics Corporation, Singapore) as previously described (26). These samples were selected to assess the seromic profile associated with the period of BCG treatment and that of ipilimumab treatment separately. The array contains quadruplicate spots of 1,627 full-length, correctly folded, and fully functional immobilized self- and cancer proteins. These include cancer antigens [mainly cancer-testis antigens (CTAs)], transcription factors, kinases, signaling proteins, and others (Table S1 in Supplementary Material). Given the expected reactivity toward self-antigens, 19 healthy individual samples were independently assayed as controls. Raw data were processed and normalized using a robust pipeline that has been previously described (27) (Supplementary Methods). Statistical analysis (GraphPad Prism and Microsoft Excel) and data-clustering methods [Multiple experiment Viewer (MeV)] were then applied to the resulting data and visualized using above significance threshold counts, box plots, comparative size-proportional pie charts, dendrogram, and heat maps.

## Results

### Baseline Patient Characteristics

Between April 2014 and June 2015, five patients were enrolled in the study. Cohort 1 included patients 1–3 in group 1 who received a 0.16–0.64 × 10^6^ cfu BCG dose and patients 4 and 5 in group 2 who received a 0.80–3.20 × 10^6^ cfu BCG dose. No patients were enrolled in planned group 3 or cohort 2. The mean age of patients was 59 years (range 43–71 years), with three males (60%). Two patients had a *BRAF* mutation and one patient a *NRAS* mutation. Two patients had prior immunotherapy with an NY-ESO-1 vaccine or pembrolizumab, and one patient had undergone prior targeted therapy with a BRAF inhibitor (Table 1).

**Table 1:** Baseline patient characteristics.

Patient	Gender	Stage (AJCC v7)	Mutational status			Prior systemic treatment (Y/N)	First-line treatment	Second-line treatment
			*BRAF V600*	*NRAS*	*c-KIT*			
1	M	M1b	WT	unknown	unknown	N	NA	NA
2	F	M1c	WT	L52V	WT	Y	NY-ESO-1 vaccine	NA
3	M	M1c	V600E	WT	WT	Y	BRAF inhibitor (PLX3603)	NA
4	F	M1a	V600K	WT	WT	N	NA	NA
5	M	M1b	WT	WT	WT	Y	pembrolizumab	NA

*M, male; F, female; WT, wild-type; N, no; Y, yes; NA, not applicable; AJCC v7, American Joint Commission on Cancer classification, version 7*.

### Safety

All patients experienced treatment-related adverse events (Table 2). Within cohort 1, group 1 patients displayed grade 1 adverse events, including fatigue, nausea, and pruritus. One patient displayed minor discomfort in the injected lesion site. Both group 2 patients in the BCG dose-escalation cohort also displayed grade 1 events, including pruritus and fatigue, but additionally these patients experienced high-grade irAEs, patient 4 with grade 3 pruritus, rash, and hepatitis on D49, and patient 5 with grade 4 colitis and secondary grade 3 small bowel ileus on D94 (Figure S1 in Supplementary Material). These irAEs were of high grade at the first onset and led to the discontinuation of the study.

**Table 2:** Administered treatment schedule, clinical responses, adverse events, and subsequent treatments across cohort.

Patient	Cohort 1	*Bacillus Calmette–Guérin* (BCG) dose	Site(s) of BCG injection	Number of ipilimumab doses received	Response		Sites of progression	irAEs	Other AEs	Subsequent treatment
					Injected lesions	Noninjected sites				
1	Group 1	0.16–0.64 × 10^6^ cfu	Left axilla^a^	4	PD—required resection	PD	Abdominal wall, pelvic node	G1 pruritus	G1 fatigue, G1 left axillary discomfort	Nivolumab

2	Group 1	0.16–0.64 × 10^6^ cfu	Right axilla^b^	4	SD	PD	Bone, liver	0	G1 fatigue, G1 nausea	Nivolumab

3	Group 1	0.16–0.64 × 10^6^ cfu	Left axillary nodule^a^	2	SD	PD	Pulmonary, pleura, bone, and hepatic	0	G1 fatigue	Dabrafenib and trametinib

4	Group 2	0.80–3.20 × 10^6^ cfu	Right upper thigh^a^	1	NE	NE	NE	G1 diarrhea, G3 pruritus, G3 rash, G3 hepatitis	G1 nausea, G2 fatigue	Dabrafenib and trametinib

5	Group 2	0.80–3.20 × 10^6^ cfu	Right anterior chest wall, right shoulder^a^	3	PD	PD	Cutaneous lesions	G1 pruritus, G1 rash, G2 diarrhea, G3 small bowel ileus, G4 colitis	G1 fatigue	DTIC

*irAEs, immune-related adverse events; AEs, adverse events, PD, progressive disease; SD, stable disease; NE, not evaluated; Gn, grade n*.

*^a^Subcutaneous*.

*^b^Lymph node*.

### Clinical Activity

Injected lesions progressed in two patients (one resection required, Figure S2 in Supplementary Material), remained stable in two others, and was not evaluated in one patient (taken off study on D49). No clinical responses were observed at noninjected sites of disease; four patients had progressive disease on the basis of RECIST v1.1 criteria and irRC (Figure S3 in Supplementary Material). The remaining patient was taken off study early and did not have tumor measurement assessments. All patients ultimately progressed at sites that included lung, liver, bone, and skin.

### Treatment-Induced Changes in the Autoantibody Repertoire

Plasma samples from five patients were analyzed for autoantibody responses by Immunome™ protein array at three distinct available time points: pre-BCG, post-BCG, and post-ipilimumab. Minor variations in sampling time point between patients are negligible, as changes in the autoantibody repertoire remain detectable for at least 90 days, due to the sensitivity of the assay and the expected 30-day half-life of specific autoantibody titers. In addition, plasma samples from 19 anonymized healthy individual sera were assayed to establish antigen-specific significance thresholds. Visual assessment of all slides revealed high-quality printing and slide handling. Data resulting from seven antigens (CASP10, COMMD3, FANCG, SMARCE1, STAT1, TNFRSF11B, and TYR) were excluded from analysis, because replicates were flagged as “noisy” on all slides. High levels of saturation were repeatedly detected against RBPJ for all patient samples, most likely due to its specific binding to the immunoglobulin kappa-type J segment recombination signal sequence. Nonetheless, this antigen was not excluded from analysis. Positive control CV calculations revealed a variation of 3.4% (cy3-biotin-BSA spots), not requiring data normalization to be performed. The remaining average net intensity autoantibody data against 1,620 antigens was used for downstream analysis. Significance thresholds were calculated as 2 SD from the mean of the data derived from 19 healthy individuals, on an antigen-by-antigen basis (Table S2 in Supplementary Material).

**Figure 2:**
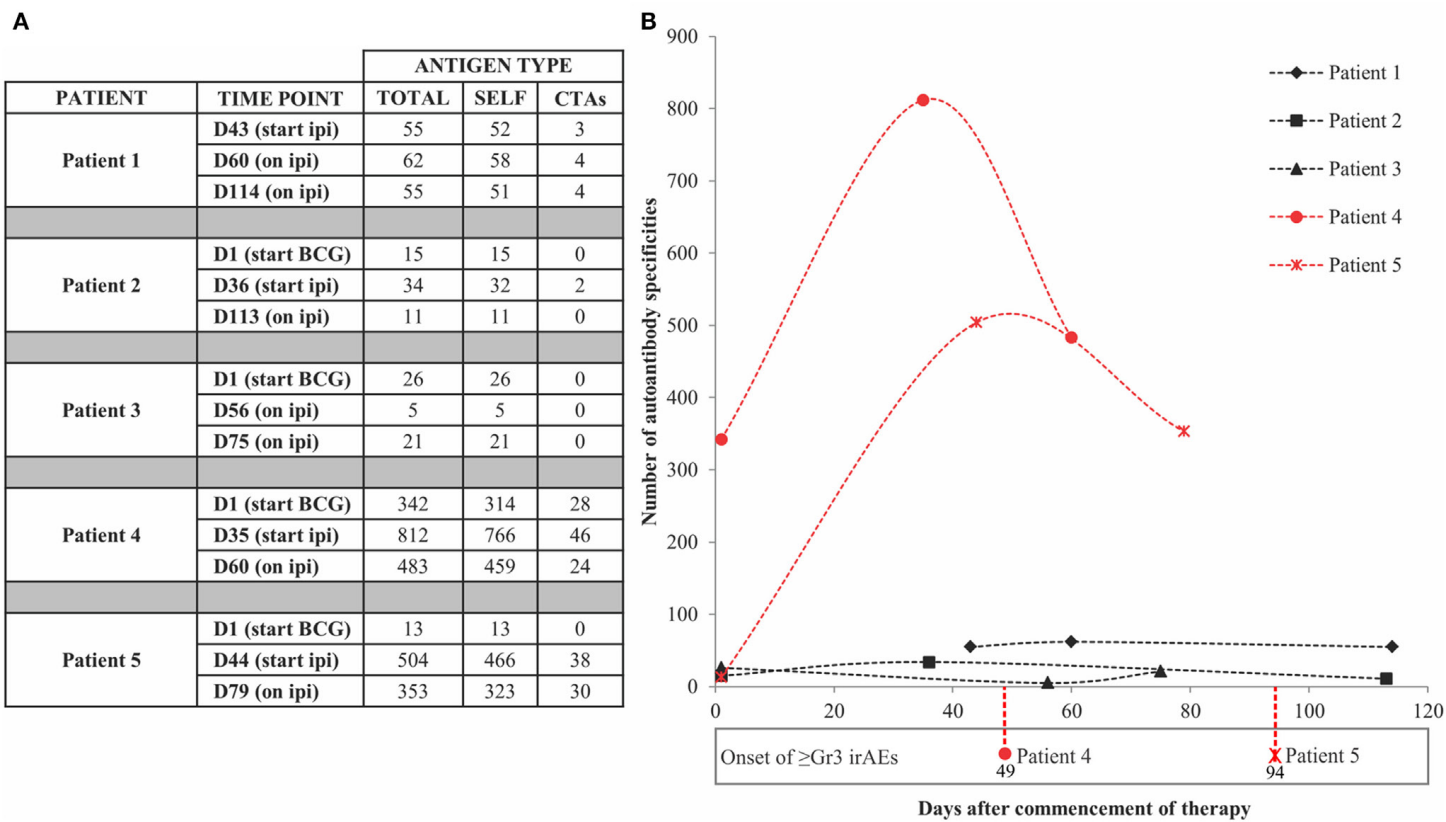
Antigen-specific autoantibody counts above healthy individual-derived thresholds of significance. **(A)** Tabulated counts divided by self-antigens (SELF) and cancer-testis antigens (CTAs). **(B)** Plotted counts comparing patients who developed high-grade immune-related adverse events (irAEs) (red) versus those who did not (black), along with the day of onset of high-grade irAEs. Dn, day n; Ipi, ipilimumab; Gr3, grade 3.

**Figure 3:**
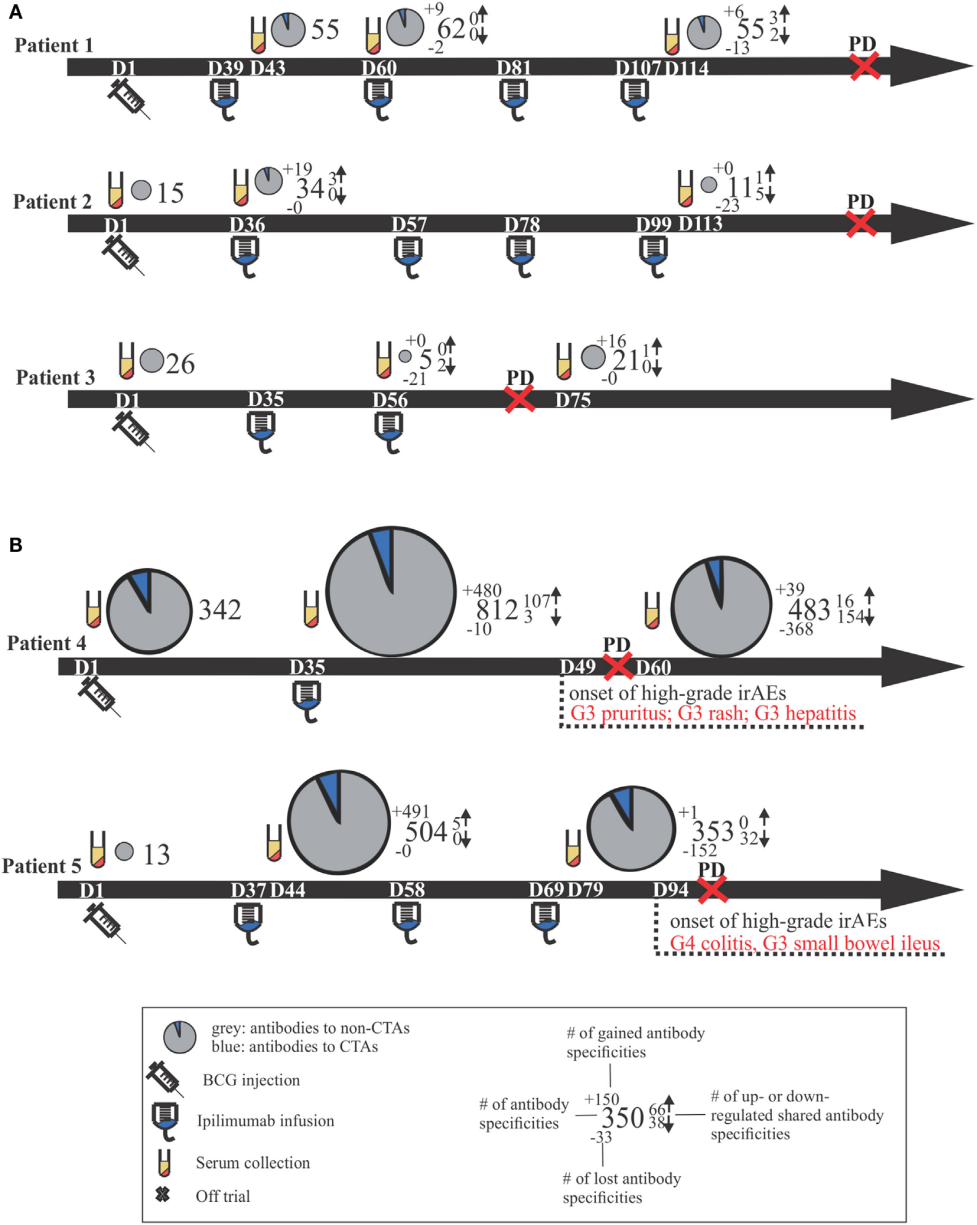
Clinical time lines for all patients, including comparative size-proportional pie charts representing the number of antigens toward which antibody titers were detected. Time lines are separated by patients who did not develop high-grade irAEs **(A)** versus those who did **(B)**. Dn, day n; PD, progressive disease; CTAs, cancer-testis antigens; Gn, grade n; irAEs, immune-related adverse events.

Positive signals above these thresholds and the day of onset of high-grade irAEs are shown in Figure 2. Although normally expressed only in the human germ line, CTAs are aberrantly overexpressed in cancer (28) and enable the assessment of cancer-specific responses alongside the broad autoantibody responses that may be unrelated to cancer immunity. Autoantibody responses according to clinical time lines are shown in Figure 3. Patients 1 (*n* = 71/1,620), 2 (*n* = 34/1,620), and 3 (*n* = 27/1,620) developed response to relatively few antigens over the period of study. This averaged 3% of the array content. By contrast, patients 4 (*n* = 853/1,620) and 5 (*n* = 505/1,620) had a substantially larger amount of *de novo* and induced counts. These represented an average of 42% of all antigens on the array. This increase was equally evident for both self- and cancer antigens. It is worth noting that enhanced autoantibody reactivity was only seen in the patients who experienced high-grade irAEs (Figure 3B). Furthermore, this broad repertoire was either preexisting at baseline (patient 4) or induced after BCG administration (patient 5) and preceded the development of symptoms in both instances.

**Figure 4:**
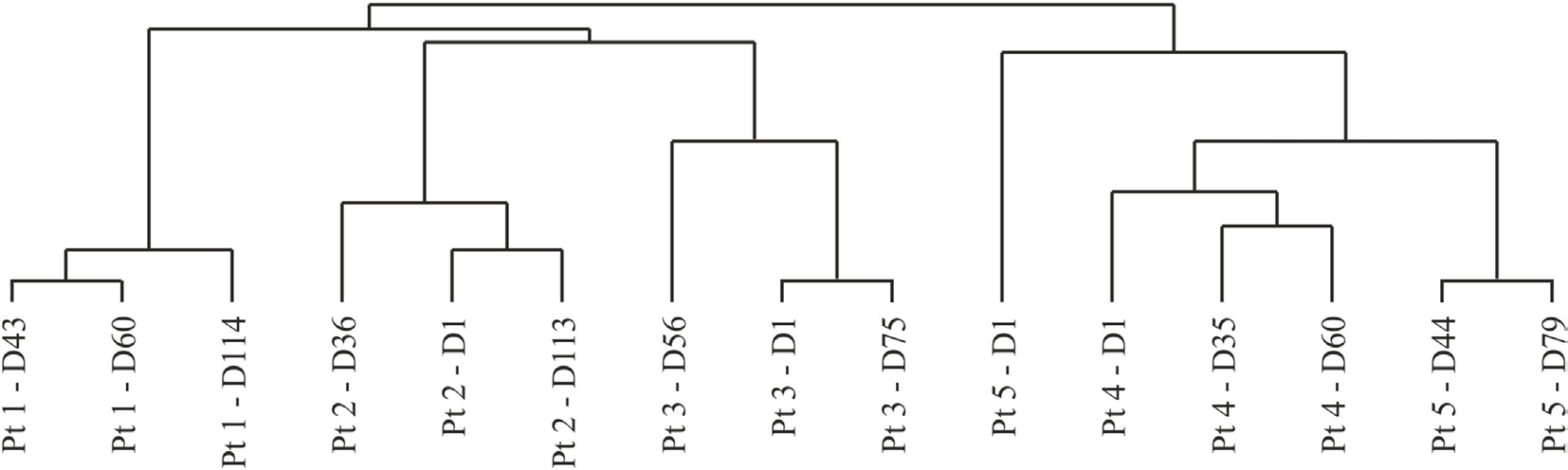
Dendrogram resulting from hierarchical clustering using the Spearman rank correlation method with average linkage. Dn, day n; Pt, patient.

In addition, the resulting data were analyzed using the Spearman rank correlation, as a means of assessing sample clustering. When inspecting the resulting dendrogram (Figure 4), all patient time points clustered together on a patient-by-patient basis, serving as an internal validation of the resulting data. Two distinct sample clusters were apparent, adequately separating patients displaying low- and high-grade irAEs. The baseline data for patient 5 were distinct from the remaining time points and from patient 4, which again highlighted that the boost in excessive autoantibody reactivity only occurred after BCG administration, rather than a pretreatment existing enhanced reactivity. Autoantibody profiles did not overlap between patients, with unique antigen targets being detected in each patient. In addition, despite the high-grade irAEs being reported in two different organs, the liver and bowel, it is unclear whether there are apparent organotypic differences in the autoantibody repertoires of these patients due to limited patient numbers.

## Discussion

Clinical trials, which combine IL immune-stimulants with ICI, aim to extend the effectiveness of cancer immunotherapy by recruiting responses against additional cancer antigens within the injected tumor. This is the first such trial to evaluate BCG, a historically validated IL therapy, with ipilimumab in patients with advanced melanoma. Although IL BCG was well tolerated, those patients treated with the higher dose of BCG (0.80–3.20 × 10^6^ cfu) developed high-grade irAEs following ipilimumab. All irAEs were typical of ipilimumab and managed as per standardized treatment algorithms. We propose that BCG may have contributed to enhanced immune activation and therefore higher-grade irAEs. These irAEs together with the availability of anti-programmed death 1 (PD-1) therapies as first-line therapy at our institution resulted in slow patient enrollment and early termination of the trial; however, our preliminary correlative seromic analyses of patients with severe irAEs suggest the potential value of serology as a diagnostic tool to anticipate and evaluate autoimmune toxicity.

As the treatment of advanced melanoma continues to evolve, combination and/or sequential treatments with immunotherapy, targeted therapy, chemotherapy, radiotherapy, and even surgery are being trialed to harness an antitumor immune response to obtain maximum clinical benefit. Talimogene laherparepvec is the first oncolytic virus approved by the U.S. Food and Drug Administration for the local treatment of unresectable lesions in patients with recurrent melanoma. Approval was based on the phase III study, OPTiM, which demonstrated significant improvement in responses in injected and uninjected lesions versus subcutaneous GM-CSF (29). Recent studies combining this oncolytic virus with pembrolizumab have demonstrated high overall (62%) and complete response (33%) rates in advanced melanoma (30). Ipilimumab has been evaluated in combination with vaccination (31, 32). In one study (31), ipilimumab in combination with a peptide vaccine resulted in two complete responses and five partial responses among 56 patients with progressive stage IV melanoma, with each of these responses shown to be durable. In another trial (32), patients with resected stage IIIC/IV melanoma received ipilimumab plus a multipeptide vaccine; 25% of patients had grades 3–4 irAEs that were dose-limiting and 27 of 75 patients relapsed after a median follow-up of 23 months. While several such combinations of localized plus systemic immunotherapies offer promise, many unselected patients with a higher disease burden or more rapidly progressive disease still face poor outcomes with existing immunotherapy combinations.

The onset of irAEs resembling classic autoimmunity remains a limitation in cancer immunotherapy (33), and while generally manageable when immunotherapeutics are administered as single agents, the incidence can increase substantially when immunotherapeutics are combined (34). Developing strategies that rationally combine immunotherapeutic agents to minimize toxicities and maximize efficacy is therefore an important area of ongoing investigation. While the identification of predictive biomarkers for therapeutic efficacy is actively being pursued (35–39), comparatively little attention has been placed on identifying reliable biomarkers that can predict adverse autoimmune events.

In this study, we investigated the autoantibody repertoire of a small number of trial patients using the Immunome™ protein array that can identify serological responses to over 1,600 human proteins. We found that the two patients who developed clinically severe autoimmunity had an accompanying profound serological signature, reflecting immune reactivity against a broad panel of autoantigens. Indeed, patients experiencing high-grade irAEs could readily be distinguished on the basis of these autoantibodies which were reactive against almost half of the proteins on the array. Importantly, these elevated autoantibody specificity counts preceded the development of clinically evident autoimmunity. Despite the very limited patient numbers in this pilot trial, it is tempting to speculate that measuring the autoantibody repertoire of cancer patients at risk of experiencing irAEs may predict the development of toxicity.

The breadth of this autoimmune reactivity was suggestive of a systemic B cell deregulation, rather than a focused immune response, as might typically occur following an infection or vaccine. Similar responses have been reported in chronic humoral rejection of organ transplants (40). We postulate that the chronic inflammatory conditions associated with interstitial mycobacterial infection resulted in the indiscriminate release of both cancer antigens and autoantigens that were not cancer-specific. In the presence of an ICI, both anticancer immunity and autoimmunity were enhanced. This is in accordance with the proposed notion that the disruption of immune tolerance and the onset of inflammation can enhance autoantibody production (41). There was no sign of clinical activity, so the reactivity against cancer-specific targets did not appear to be clinically useful; however, with a small number of patients, it is difficult to be certain. This specific immunotherapy combination will not be pursued further due to the apparent excess of clinical toxicity. Nonetheless, larger studies—likely in the setting of more tolerable immunotherapy combinations—will be required to validate these seromic findings and to better understand the involved mechanisms.

In conclusion, dose escalation of IL BCG followed by ipilimumab therapy in a pilot trial of limited patient numbers was not well tolerated in advanced melanoma patients and showed no evidence of clinical benefit. Whether the onset of the observed high-grade irAEs was enhanced by IL BCG or simply a result of ipilimumab alone remains unclear. Nonetheless, investigating humoral immunity may offer a means to detect the early onset of a wide spectrum of irAEs in cancer patients treated with immunotherapy, warranting further larger studies.

## Ethics Statement

This study (ClinicalTrials.gov number NCT01838200) was conducted at Austin Health, Heidelberg, Australia, and was approved by the institution’s human research ethics committee. Written informed consent was obtained from all patients, and all methods were performed in accordance with the protocol-specified guidelines and regulations.

## Author Contributions

JD, AB, and JC were responsible for the immunome array study design. JD was responsible for the data analysis, representation, and interpretation. JB, AB, and JC provided additional expert input into the key findings. MA and JC were responsible for the planning and execution of the clinical trial. SP and MA provided relevant clinical information. KW, AP, CT, and SO were responsible for clinical sample processing and additional immunology experiments that were excluded from this article. JD wrote the manuscript, and SP, MA, JB, AB, and JC reviewed and edited. All authors read and approved the submitted version.

## Conflict of Interest Statement

JB is a consultant for Sengenics and developed their Immunome™ protein array product while being an academic researcher at the University of Cambridge. No additional authors have a conflict of interest to declare.
