# Supplementary material for: Autoantibodies May Predict Immune-Related Toxicity: Results from a Phase I Study of Intralesional Bacillus Calmette–Guérin followed by Ipilimumab in Patients with Advanced Metastatic Melanoma
Source: Front Immunol. 2018 Mar 2;9:411. doi: 10.3389/fimmu.2018.00411 (PMC5840202; doi:10.3389/fimmu.2018.00411)
Supplement: Supplementary file 3 [file data_sheet_1.docx]

***Supplementary Material***

**Autoantibodies may predict immune-related toxicity: results from a phase I study of Intralesional Bacillus Calmette-Guerin followed by ipilimumab in patients with advanced metastatic melanoma**

**Jessica Da Gama Duarte, Sagun Parakh, Miles C. Andrews, Katherine Woods, Anupama Pasam, Candani Tutuka, Simone Ostrouska, Jonathan M. Blackburn, Andreas Behren*, Jonathan Cebon***

*Correspondence: Corresponding Authors: [andreas.behren@onjcri.org.au](mailto:andreas.behren@onjcri.org.au), [jonathan.cebon@onjcri.org.au](mailto:jonathan.cebon@onjcri.org.au)

**1 Supplementary Tables**

**Table S1. Immunome^TM^ protein array list.**

Please view attached excel data sheet.

**Table S2. Pooled healthy donor-derived significance thresholds on an antigen-by-antigen basis.**

Please view attached excel data sheet.

**2 Supplementary Methods**

**Detailed protein array data processing method.**

The data processing and normalization pipeline used included:

1) Using the median net intensity (median foreground intensity – median background intensity) values for each spot, all replicates displaying 75% of pixel intensities below the local median background threshold (% > background + 2 x standard deviation) were flagged as “noisy” and discarded;

2) All replicates exhibiting saturating pixels above 5% (dust particles) were flagged as “saturated” and discarded;

3) If 3 or more replicates per antigen are discarded via flagging as “noisy” or “saturated”, the corresponding antigen was excluded from further analysis. The only exception being where saturation levels are above 75% across all antigen replicates (real saturation due to scanner’s maximum signal detection capacity). When using the quantitative array data in downstream analysis, slides displaying this level of high saturation across a subset of antigens require re-scanning at a lower gain setting;

4) The coefficient of variation (CV) of all remaining antigen replicates was calculated, and any outlier replicate(s) that increase the CV above 20% were flagged as “high CV” and discarded;

5) The average of all unflagged net intensities was calculated for each antigen in all instances where at least duplicates were available;

6) The CV of all replicate cy3-biotin-BSA positive controls within each slide was calculated, and compared across slides. If the inter- and intra-slide CVs were above 5%, the data required normalization using a composite method previously described (Duarte, Serufuri et al. 2013).

**3 Supplementary Figures**

**
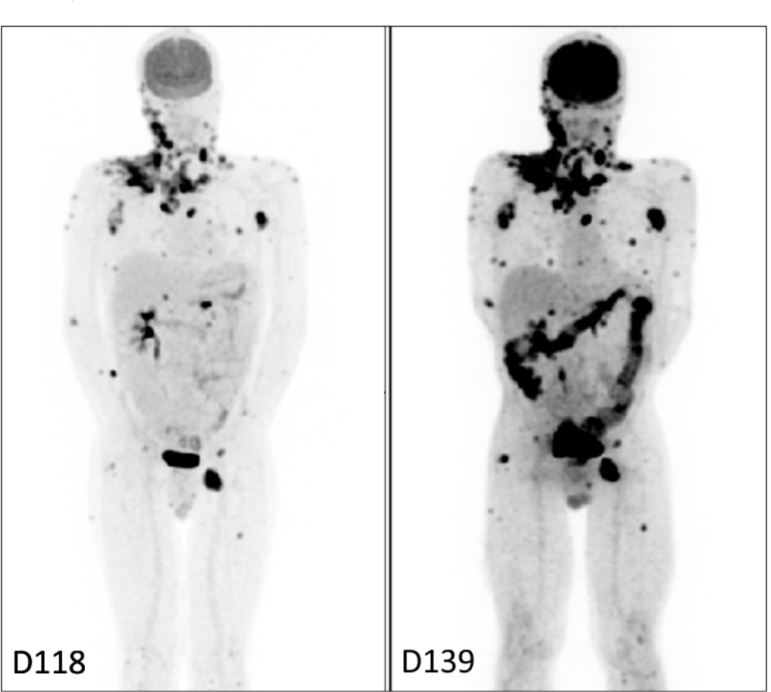
**

**Figure S1. Whole-body PET images taken on D118 (left panel) and D139 (right panel) demonstrate suspected treatment-induced grade 4 colitis in patient 5.** Widespread disease progression is also evident. Dn – day n.


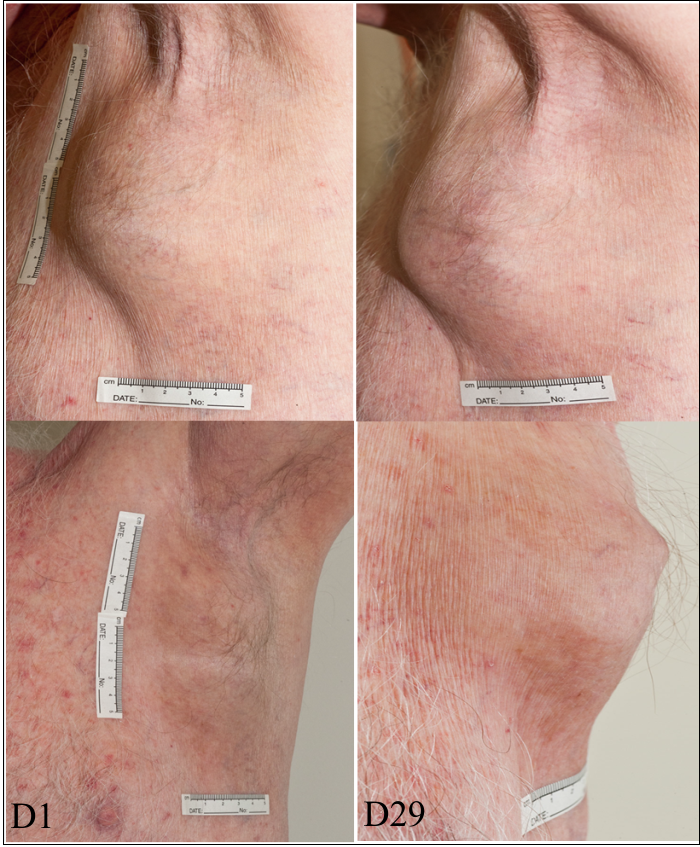


**Figure S2. Clinical photographs taken of the left axilla (injected lesion) of patient 1 on D1 (left panel) and D29 (right panel) with evident progression.** This lesion subsequently required surgical resection. Dn – day n.


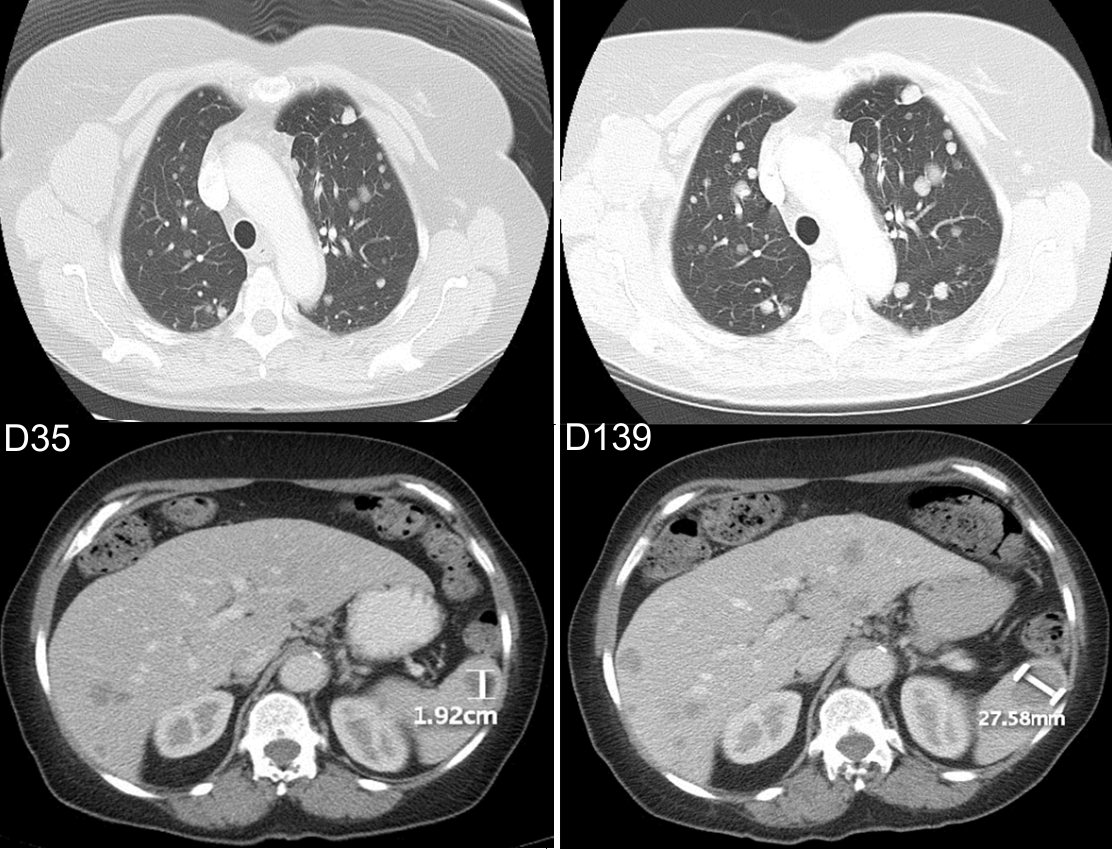


**Figure S3. CT scans of the lungs (upper panel) and spleen (lower panel) of patient 2 on D35 and D139 showing the progression of target lesions.** Dn – day n.
